# A Review of Thermal Detectors of THz Radiation Operated at Room Temperature

**DOI:** 10.3390/s24216784

**Published:** 2024-10-22

**Authors:** Zbigniew Bielecki, Janusz Mikolajczyk, Jacek Wojtas

**Affiliations:** Institute of Optoelectronics, Military University of Technology, S. Kaliskiego Str. 2, 00-908 Warsaw, Poland; zbigniew.bielecki@wat.edu.pl (Z.B.); jacek.wojtas@wat.edu.pl (J.W.)

**Keywords:** terahertz technology, thermal detectors, THz detectors

## Abstract

This article concerns optical detection issues in the terahertz (THz) range. This is a kind of guide to various types of uncooled thermal detectors in the most often applications. Particular attention is paid to the principle of their operation, technology, and practical features. In addition, some detection methods were also characterized by comparing their performances. The article ends with a performance summary of the selected THz thermal detectors.

## 1. Introduction

**Figure 1:**
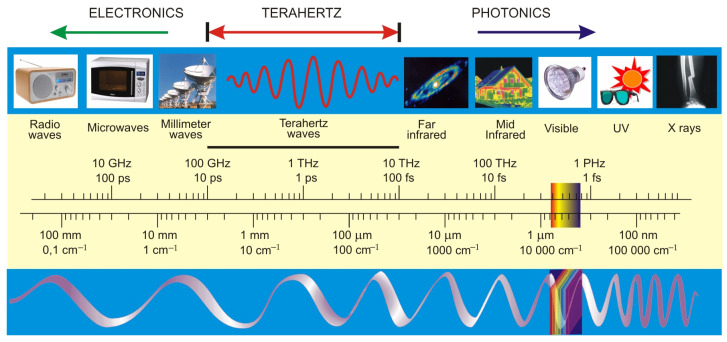
The electromagnetic spectrum [1].

Electromagnetic radiation known as the terahertz (THz) range (Figure 1) is considered the least explored range of optical radiation and poses a challenge to both science and the development of technologies in electronics and photonics. It is situated between infrared light and microwave radiation with a spectral region in the frequency range from 0.1 THz to 10 THz (wavelengths from 30 µm to 3 mm). It is partly overlapping with a loosely treated submillimeter (sub-mm) band of (0.1–3) THz.

**Figure 2:**
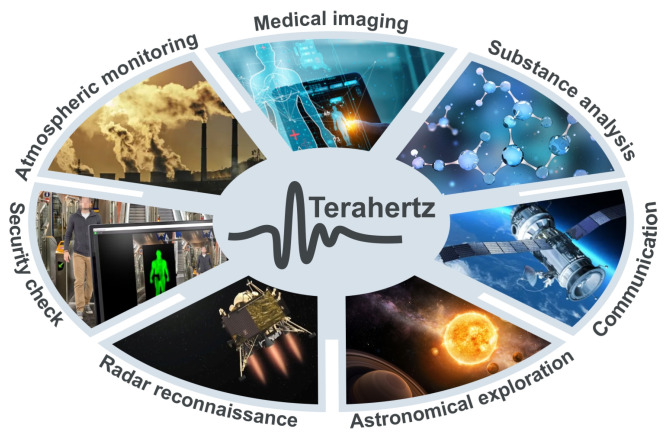
Scheme of explored and future THz applications [2].

Although THz technology, in general, is still in the early stage of development, many potential applications have been defined, e.g., in astronomy, communications, defense, security, industry, material studies, and medicine. For example, it is used for breast cancer diagnostics, colon cancer, burn imaging, etc. (Figure 2).

In practice, THz radiation penetrates a variety of dielectric materials, such as plastics, papers, and many organic compounds, but not liquids and metallic objects. Due to low photon energies, it is not harmful to biological tissues, including human bodies. Moreover, a lot of biological and chemical materials have unique “spectral fingerprints” within this spectral range [3,4].

The application area of THz radiation causes a dynamic development of its detection and imaging technology. This development is described in the literature as ongoing. There are many review articles and even books on this topic. The analyzed area of these technologies often covers a vast spectrum of THz radiation detectors and indicates the directions of their possible development. However, potential users often only look for ready-to-work commercial devices they could use in their applications. The article’s primary goal, and its unique achievement, is to provide a comprehensive review of these devices, considering the preliminary analysis of their principle of operation and description of selected technologies. The review concerns only the group of thermal detectors, which is an initial selection of knowledge about THz detecting devices for a potential user.

## 2. Materials and Methods

### 2.1. Terahertz Detector Classification

**Figure 3:**
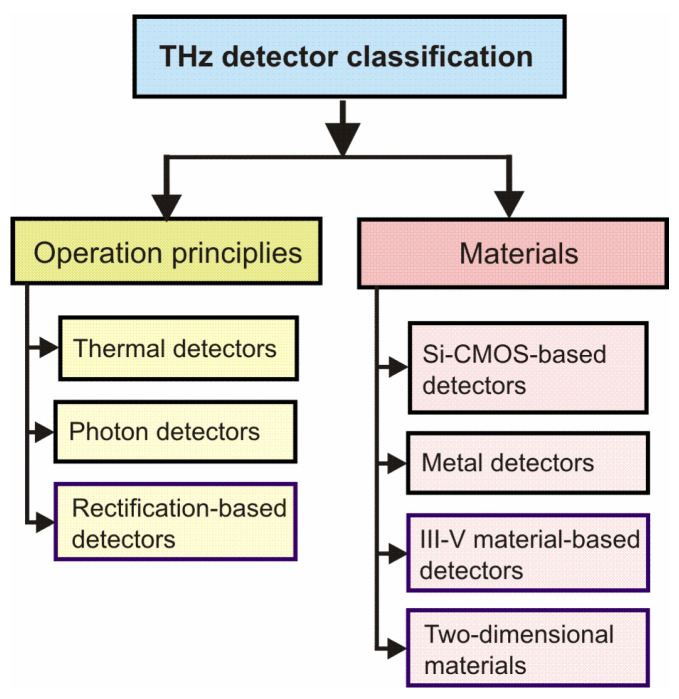
Classification of THz detectors (adopted after [5]), where CMOS is a complementary metal–oxide–semiconductor.

THz detectors can be classified based on operation principles, used materials, and applied measurement methods (Figure 3).

The first classification of THz detectors is based on the operation principle, including thermal, photon, and rectification detectors [1]. In photon detectors, the radiation is absorbed within the material by interaction with electrons either bound to lattice and impurity atoms, or free electrons. The electrical output signal results from the distribution change in electronic energy. The photon detectors class is subdivided into different types depending on the interaction’s nature. The most important are intrinsic detectors, extrinsic detectors, photoemissive detectors (Schottky barriers), as well as quantum well and superlattice detectors. There are many literature references devoted to the theory and technology of optical radiation detectors, working in different ranges of the electromagnetic spectrum, for example, Refs. [1,6].

**Figure 4:**
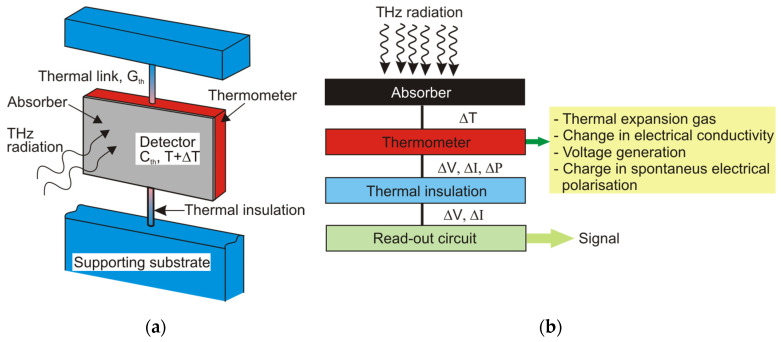
(**a**) Thermal detector: scheme of operation [8]; (**b**) the main steps of the detection procedure.

Generally, a thermal detector contains a thermometer and a thermal link (Figure 4a). The thermometer itself combines a radiation absorber and a temperature transducer. Incident radiation causes a temperature rise in the thermometer (ΔT) with a change in some of its physical properties (Figure 4b). This change is processed by a read-out circuit [7].

**Figure 5:**
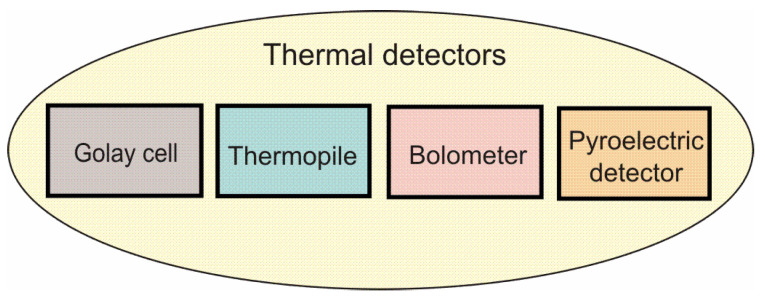
Thermal detectors used for THz detection.

Thermal detectors can be subdivided into Golay cells [9], thermocouples/thermopiles [10], bolometers [11], and pyroelectric detectors [12] (Figure 5).

For the thermal link, usually, thermal conductance *G*th is very crucial. The construction of the thermometer should provide high absorption, low reflection, low heat capacity (*C*th), and low electrical noise [13,14]. The radiation absorption can be simply elevated by increasing the absorber thickness, but higher heat capacity causes a slower detector response. The application of a special trap design resolves this problem. Table 1 shows the most-used absorber materials for THz applications [15].

THz detection methods are divided into two groups: direct detection methods, and heterodyne detection ones. The first one allows only THz signal amplitude detection, whereas heterodyne systems enable the determination of the amplitude and phase of the signal.

In the direct detection method, the photodetector converts the THz radiation into an electrical signal, which is amplified by a preamplifier. The preamplifier must have a low self-noise level and a frequency band so as not to distort the shape of the input waveforms. The sensitivity of the THz detection system depends primarily on its first stage, i.e., the detector and preamplifier.

**Figure 6:**
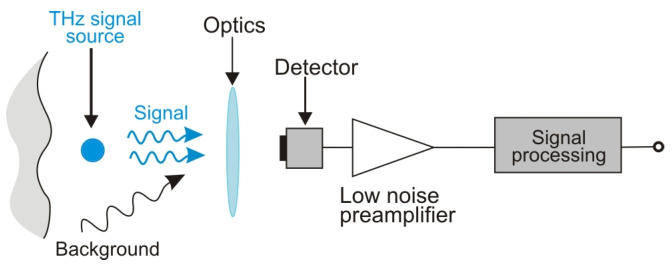
A simplified schematic of the direct detection method.

Figure 6 shows a simplified diagram of the direct detection system. In this design, it is necessary to minimize the noises from various sources, i.e., background, detector, preamplifier, and any signal processing unit. If further noise minimization of the system is not possible, advanced methods of THz detection can be used (e.g., lock-in) [16,17].

In fact, in the last decade, CMOS and SiGe HBT technology has significantly advanced terahertz (THz) applications. These technologies have shown great promise in reducing the cost of THz systems and enabling various applications [18].

### 2.2. Figures of Merit

As was already indicated, the performance of the terahertz detectors depends on radiation absorbance, the heat capacity of the absorber, the thermal conductance between the heat source and heat sink, and the measuring accuracy of the temperature rise. These factors are defined not only by the material parameters used in the detector’s design but also by its geometrical construction. The performances of terahertz detectors are evaluated based on the following criteria:

- Noise-equivalent power (*NEP*) is the incident power on the detector that produces at its output a signal-to-noise ratio of unity. The *NEP* is also determined for a defined reference bandwidth which is usually assumed to be 1 Hz. The lower the *NEP* value is, the better the sensing limit of the detector;
- Responsivity is defined as the ratio of the root mean square (RMS) value of the detector’s electrical output signal to the incident radiation power. At the visible and near-infrared range, most thermal detectors have spectrally flat responsivity, whereas above >300 µm the responsivity is low. The higher the responsivity of the detector, the lower changes in the THz radiation can be detected;
- Detectivity (*D*) is the reciprocal of *NEP*, but a more useful parameter is the normalized detectivity D*, which was introduced by R.C. Jones as a function of the signal bandwidth and detector active area. For thermal detectors, D* varies from 10^7^ to 10^10^ cm√Hz W^−1^. In practice, the detectivity must be as large as possible;
- The time constant (τ) of the detector is determined by the heat capacity (*C*th) and the thermal conductance (*G*th). It expresses the speed at which a device responds to a change in incident power. To obtain a fast detection system, these thermal parameters should be as small as possible. The time constant ranges from a few milliseconds to seconds.

## 3. Results

### 3.1. Comparison of Uncooled Thermal Detectors

**Figure 7:**
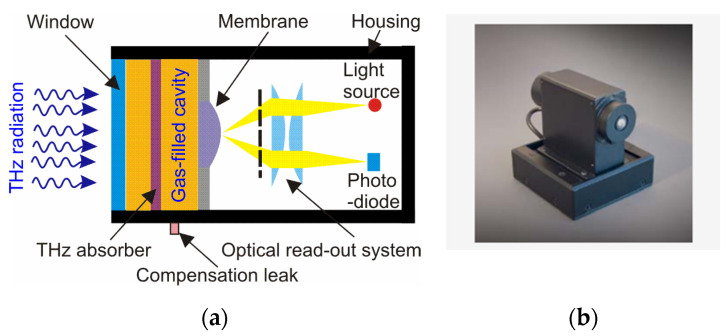
(**a**) The schematic principle of Golay cell [19]; (**b**) photo of Golay detector [20].

The Golay cell is one of the oldest designs of THz detectors. As shown in Figure 7, the core part of the Golay detector is a gas-filled cavity. There are usually selected argon or xenon due to their low thermal conductivity. One wall of the cavity is equipped with a THz absorber, and the other wall of the cavity is a flexible membrane with a mirror.

**Figure 8:**
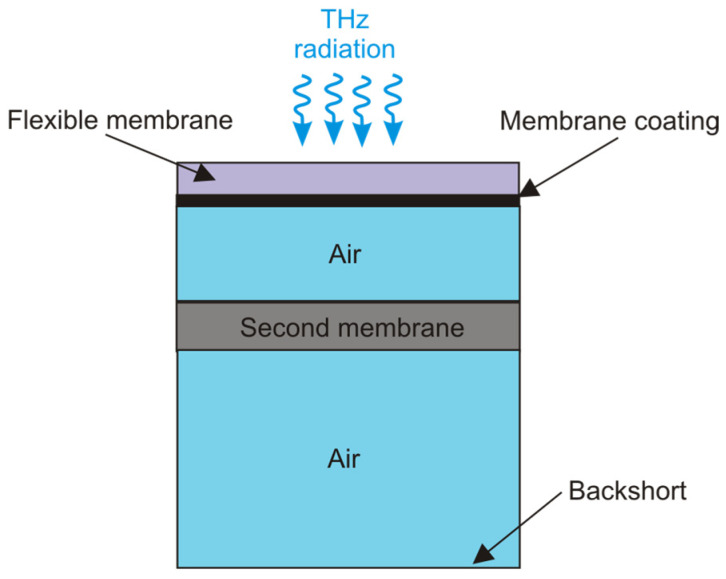
Cross-section of the Golay detector structure [22].

The absorbed THz radiation increases the heat of the gas expanding the flexible membrane with a mirror. Light from the light-emitting diode (LED) is directed onto this mirror, which is then reflected and detected by a photodiode (PD). The motion of the membrane produces a change in the photodiode output signal. These devices for the THz radiation are characterized by reasonable performances, e.g., typical responsivity of approx. 1.5 × 10^5^ V/W at 1 THz and noise equivalent power of about 1.2 × 10^−10^ W/Hz^1/2^ [21]. The advantage of this type of detector is a flat spectral response over a wide wavelength range. The Golay detectors with polyethylene windows are very sensitive (sub-nW) and operate at room temperature within a range of 20–1000 μm [9]. The main deficiencies include, e.g., the large sizes of the detector housing and power supply, a slow response time (~15 ms), a low dynamic signal range (maximum typically of 10 μW), susceptibility to being interfered with by thermal fluctuation, external radiation, and vibration, as well as a relatively high price. Moreover, these detectors include a high-density polyethylene (HDPE) or diamond window for operation, and the membrane used in the Golay cell is thin and fragile. The Golay detector has been used as a standard in extreme IR and astronomy for years. The paper [22] presents a design of a miniaturized Golay cell consisting of two absorbing membranes (Figure 8). The relative variation of capacitance of the plane capacitor formed by these two membranes deforming under the influence of THz radiation is used as a read-out. The micromachined sensor ensures a bandwidth of 0.5–4.0 THz and could be easily integrated into an array design.

**Figure 9:**
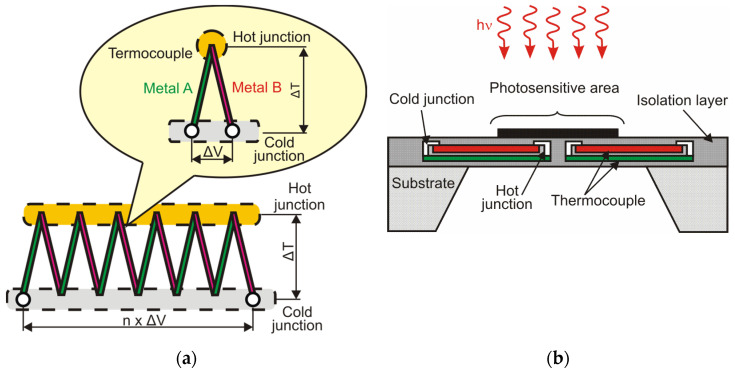
(**a**) Diagram of the thermopile; (**b**) cross-section of the thermopile with a single/dual structure [7,23].

Another type of thermal detector is a thermocouple, which is constructed as a combination of two different materials forming two junctions (Figure 9).

The detector is made by integrating the blackened radiation absorber with a measured junction. The THz radiation power *P*_abs_ is changed into the heat of the absorber. The temperature increase can be expressed by ∆*T* = *P*_abs_/*G*th. The heat changes are converted into electrical signals by using the thermocouple. The signal voltage is directly proportional to the temperature difference of the thermocouple’s junctions:

(1) $$\Delta V=\alpha_{s}\Delta T=\left(\alpha_{A}-\alpha_{B}\right)∆T,$$

where α_s_ is the effective Seebeck coefficient of materials A and B expressed in µV/K.

Thermocouples are made as single elements, with double or quadruple structures, as 16- or 32-element lines, and as mosaics with a small number of elements (e.g., eight × eight). In practical applications, a single thermocouple is not used. When *N* thermocouples are connected in series a thermopile is obtained, the responsivity of which is improved by *N* [8].

Huhn et al. demonstrated high-speed terahertz detector arrays based on thermocouples of bismuth and antimony (BiSb/Sb) coupled to antennas. Each pixel consists of eight thermocouples connected in series [24]. They operate at room temperature. The thermocouple response time was only 22 µs, and the NEP was 170 pW/Hz^1/2^.

Thermopiles operate at room temperature without the necessity of modulation of incident radiation. Even though thermopiles are not as sensitive as other thermal detectors, they are used in many applications due to their reliability and low cost, very often as standard detectors in calibrating terahertz systems.

The materials used for thermopile detectors are Ti–Al, Au–Bi, Bi–Cr, n-polySi/p-polySi, p-polySi/Al, Ti-doped Si, and Si–Ge. For example, in the case of Si, the Seebeck coefficient α_s_ is ~500 µV/K, and for metals it is about one order less. Thin film α_s_ coefficients are usually lower than bulk ones and are technology-dependent. The responsivity of such a thermopile depends on the absorber’s performance and the construction of its thermal bridges. The absorbing element can be made from thin metal films, highly doped polySi layers, metamaterials, carbon nanotubes, and metallic grids deposited on SiO_2_ membranes [25]. However, metal films must be thin enough to ensure high absorption, typically a few nanometers or even less.

**Figure 10:**
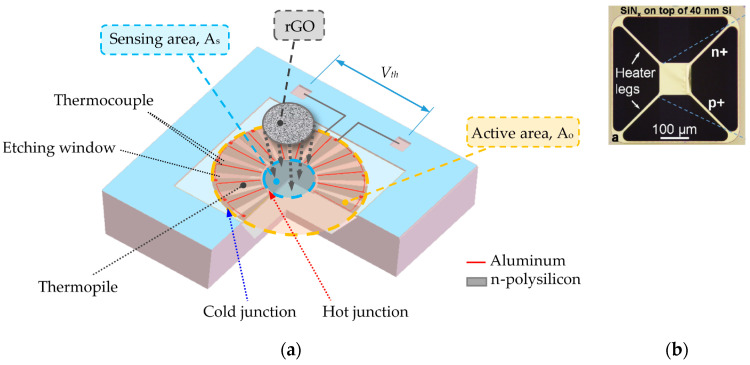
(**a**) Structure view of the CMOS–MEMS thermopile [27]; (**b**) an image of the Si nanomembrane thermal detectors [28].

For example, the pixel design of the TiN absorber and p-polySi/n-polySi thermoelement characterized by a responsivity of 28 V/W and detectivity of 1 × 10^7^ cmHz^1/2^W^−1^ at the wavelength of 118.8 µm was described in [26]. Micro-electromechanical systems (MEMS)-based thermopiles IR were also developed. Such structures were implemented to design chip technology. Using complementary metal–oxide–semiconductor (CMOS) and MEMS technology, compact, cheap, low power consumption, and high sensitivity THz detectors can be constructed. Such a structure has been designed to operate with an IR responsivity of 14.522 V/W using a reduced graphene oxide (rGO) absorber [27]. Its schematic drawing is presented in Figure 10a.

In Figure 10b, another design of uncooled nano-thermocouple detectors based on suspended 40 nm-thick single-crystalline silicon thermocouples is demonstrated [28]. The devices are micromachined using standard silicon and aluminum materials. The absence of additional micromechanical support layers minimizes the thermal conductance *G*th and enables very low noise equivalent power of 13 pW/Hz^1/2^ and quite a short response time of 2.5 ms.

**Figure 11:**
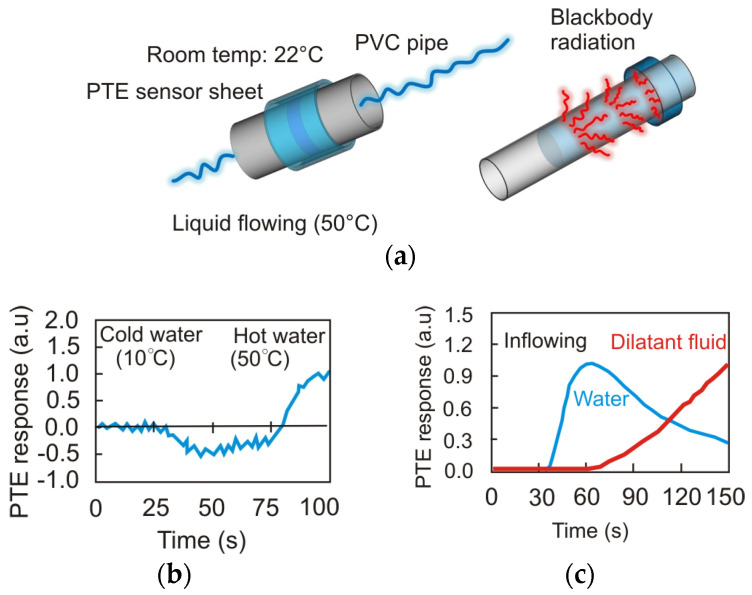
(**a**) Conceptual diagram of BBR-based PTE passive liquid monitoring with the device; device responses during liquid flowing (**b**) in different temperatures and (**c**) in different viscosities (adopted after [29]).

A development in detector design utilizing flexible electronics and optics has been evident in detector arrays. They can be used for 3D imaging and detection of broadband radiation by using a p–n junction (CNT) carbon nanotube film. Figure 11 shows a diagnostic system using an uncooled sensor with a CNT film based on the photo thermoelectric effect (PTE). The thermoelectric capabilities of these films stem from their 1D nanostructures, which enable efficient conversion.

Additionally, their remarkable ability to absorb broadband radiation, ranging from near-infrared (NIR) to the sub-terahertz (THz), contributes to their outstanding performance, even when subjected to stretching and operated at varying temperatures. Thanks to this, such CNT arrays can be used to work in particularly difficult conditions. The selection of the appropriate dopant concentration, material properties, shape, and integration technology defines their parameters. For example, with some configurations, it is possible to obtain noise equivalent power values of 236 pW Hz^−1/2^ (*λ* = 1.15 mm), 105 pW Hz^−1/2^ (*λ* = 577 μm), 30 pW Hz^−1/2^ (*λ* = 300 μm), 3.87 pW Hz^−1/2^ (*λ* = 10.3 μm), 4.5 pW Hz^−1/2^ *(λ* = 4.33 μm), and 8.68 pW Hz^−1/2^ (*λ* = 870 nm) [29].

**Figure 12:**
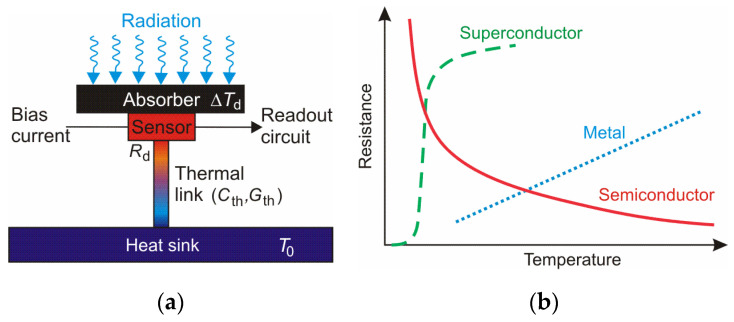
(**a**) Fundamental structure of a bolometer; (**b**) temperature dependence of resistance of three bolometer material types [30].

Another type of thermal detector is a bolometer. It is a temperature-sensitive device in which resistivity changes because of heating by absorbed THz radiation. The sensitivity of the bolometer is determined by the temperature coefficient of resistance (*TCR*), α_d_. The bolometer construction is shown in Figure 12a. It consists of an absorber and a temperature-sensitive element (sensor). The absorber should have a large radiation absorptivity and a proper size to detect signals efficiently. It can be made of a metal, a doped semiconductor, or a superconductor film [15]. Its connection to the “surroundings” through thermal conductivity should have the lowest possible value. As a result of the absorbed radiation, it is heated, and the resistance Rd changes, causing a modulation of the current flowing in the electronic circuit. The change in the bolometer resistance caused by its temperature is determined by

(2) $$\Delta R_{d}=\alpha_{d}\Delta T_{d},$$

where Δ*R*_d_ and Δ*T*_d_ are the changes in resistance and temperature of the bolometer, respectively. Figure 12b shows schematically the temperature dependence of resistance of three types of bolometer materials.

The useful bolometer should have high responsivity, low *NEP*, wide spectral range, short response time, ability for room-temperature operation, and ease of fabrication. It is difficult to obtain these parameters, parallelly, and two design configurations of these detectors can be defined: standard bolometers and microbolometers. The first ones work at room temperature with a long response time due to the demand for heating a large bulk material.

Microbolometers, on the other hand, are fabricated in small sizes on the order of tens of microns and the main challenge is to couple the incident radiation efficiently to their active area. Through the use of an antenna, the detector itself can be very small, while the antenna provides a sufficient collection of radiation. An additional advantage of the antenna coupling is its directivity, which reduces the detector response time and decreases NEP.

Bolometers can also be designed to operate as uncooled (room-temperature operation) and cooled detectors. Compared, cooled bolometers, despite their more advanced design, higher costs, and higher power consumption, provide much greater sensitivity.

The most commonly used bolometers are metal (i.e., Pt, Ti) and semiconductor ones (i.e., composition of vanadium oxide VO_x_, poly-Si and poly-SiGe alloys, Ge and amorphous Si–Ge alloys). The first group is the oldest microbolometer’s structure with *TCR* below 0.5%/K. Although metals have low *TCR*, they are characterized by low 1/f-noise increasing the signal-to-noise ratio. In practice, the resistivity of these bolometers usually decreases with growth in temperature. In the case of semiconductor bolometers, better responsivity and detectivity are obtained because of a higher value of their negative *TCR*. It is however worth noting that higher responsivity does not result in a higher value of these detectors’ detectivity. This is because they have a higher level of 1/f-noise.

The vanadium oxide thin films are used today in a large variety of bolometer products. Bolometers made of α–Si consist of very thin membranes to ensure a low thermal mass. Consequently, α–Si bolometers can benefit from a low thermal conductance while maintaining a fixed bolometer time constant.

When a sensing element acts as an absorber and thermometer, thermal detectors are said to be monolithic; inversely, detectors made of a thermometer attached to a separate absorber are referred to as composite. One of the advantages of these bolometers is that they allow optimization of each element independently.

Typical materials used for metal bolometers are nickel, titanium, platinum, niobium, palladium, or antimony. The appropriately selected materials keep stable parameters that do not strongly degenerate with operation time. For example, bismuth, which is a brittle metal, ensures good sensitivity and noise performance but causes unstable characteristics due to electromigration. Most metal bolometers are formed as film strips with a black absorber such as evaporated gold or platinum. A *TCR* of these detectors is about 0.3%/K ensuring detectivity of order 1 × 10^8^ cmHz^1/2^W^−1^ and 10 ms response time [10]. In many applications, their performance is determined by a tradeoff between speed and sensitivity. The typical *NEP* of the bolometer is very low, on the order of 3.4 × 10^−15^ W/Hz^1/2^ at 0.4 K [10] and as low as 10^−12^ W/Hz^1/2^ even at 300 K [11,31].

**Figure 13:**
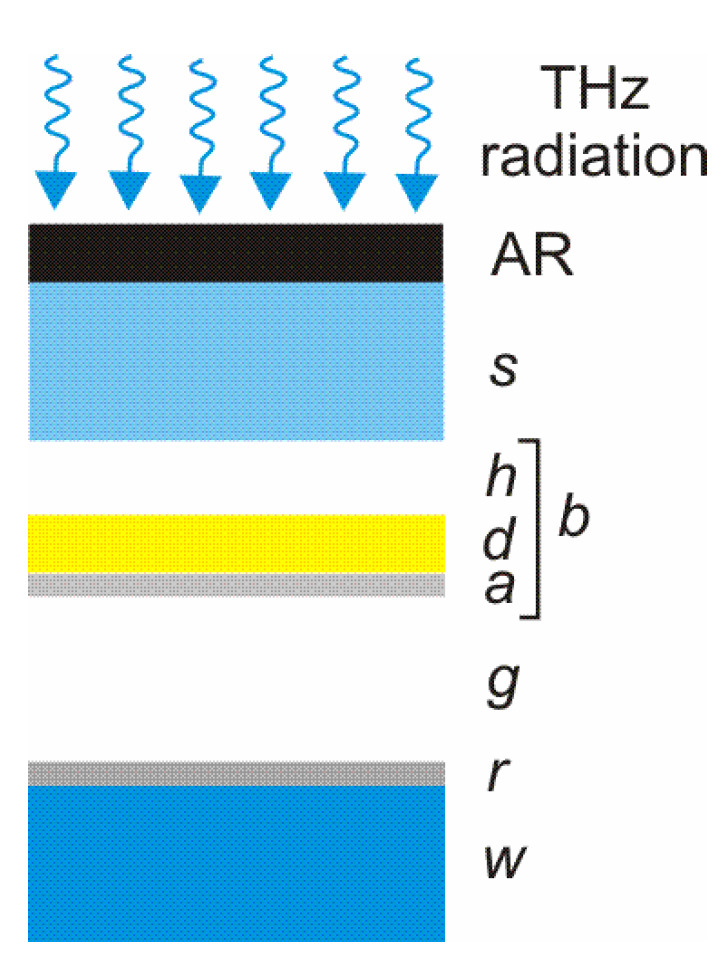
Bolometric THz detector with metallic absorber where AR is the antireflective coating, *s*—substrate, *b*—bolometer (*h*—vacuum gap, *d*—dielectric membrane, *a*—metallic THz absorber), *g*—vacuum gap, *r*—reflector, *w*—window [33].

The traditional IR bolometers are simply applied in a microbridge structure (produced by many companies, e.g., Raytheon (Arlington, VA, USA), DRS Technologies (Arlington, VA, USA), and BAE Systems Inc. (London, UK) to detect THz radiation. However, this technology does not provide so high sensitivity. Comparing levels of noise equivalent power, there are obtained values of 14 pW/Hz^1/2^ and 300 pW/Hz^1/2^ for the IR and the THz spectral ranges, respectively [32]. Figure 13 shows an example of a bolometric THz detector with a metallic absorber.

**Figure 14:**
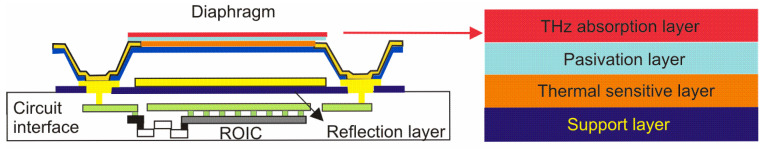
Structure of single pixel in THz focal plane array (FPA) [35].

Using an additional optical cavity (formed as a vacuum gap ‘*g*’ between the bolometer and the reflector ‘*r*’) ensures obtaining absorbance close to unity in the wavelength range of 37–200 µm [34]. In addition, such bolometer structures provide both selective and broadband THz detectors. Gou et al. [35] developed a 320 × 240 THz focal plane array with 35 mm pitch pixels (Figure 14).

The pixel is composed of a diaphragm, cell contact, and two legs to support the diaphragm. The diaphragm consists of a THz absorption layer (Ti), passivation layer (Si_3_N_4_), thermal sensitive layer (VO_x_—with *TCR*~−2.3%/K), and support layer (Si_3_N_4_), with a reflection layer placed 2.5 µm away. The reflection layer was made of NiCr thin film. To improve the rate of temperature rise (low thermal mass), the diaphragm was suspended on lags connected to the heat sink. The signal of the pixel is sent through the cell contact to readout integrated circuit (ROIC). Its basic function is to amplify and process the THz detected signal. Responsivity and *NEP* of the FPA were 2186 V/W and 45.7 pW/Hz^1/2^, respectively, for 2.52 THz radiation generated by a CO_2_ laser [35].

**Figure 15:**
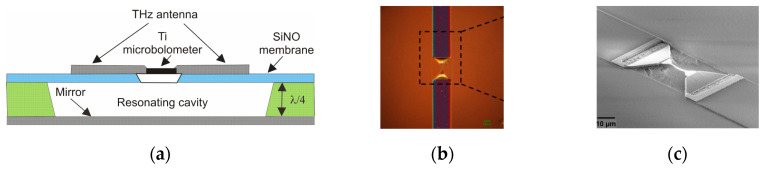
(**a**) A schematic view of the antenna-coupled Ti-microbolometer; (**b**) a flat-top view of the central part of THz antenna; (**c**) the side 3D view [38].

To enhance the absorption of THz radiation the antenna-coupled microbridge structures are used. Using antennas, the detector itself can be made very small. When the detector element is small, only a small amount of energy is required to change the bolometer resistance, so an increase in responsivity can be achieved. Well-defined antennas ensure not only very efficient coupling of radiation to detectors but also contribute to decreasing NEP, reducing the detector’s response time, or raising its operating temperature. Since the shape of the antenna affects coupling efficiency to the microbolometer, various types of antennas have been developed such as dipole, bow-tie, and spiral ones. The paper [36] presents a micro-bridge bolometer structure coupled with a spiral-type antenna. Additionally, this structure is a good solution for the mentioned method to use infrared micro-bolometer technology for THz application. Depending on the type of the antenna, the broadband or narrowband spectrum can be detected [37]. A schematic view of the antenna-coupled microbolometer THz sensor is shown in Figure 15.

In this construction, the dipole antenna and resonant-cavity design ensure spectral selectivity in the frequency range from 0.2 THz to 2 THz with maximum sensitivity at resonant frequencies of 300 GHz and 600 GHz.

However, room-temperature THz thermal detectors do not provide all the performances required in various applications, e.g., compound sensing. That is why some physical solutions for THz detection are still being developed. For example, the performed laboratory tests show that the use of MEMS or NEMS resonators is a promising approach due to fast response, high sensitivity, compactness, and miniaturization. The MEMS resonator (a microcantilever or a double-clamped beam) can be driven into resonant oscillation by an external circuit and works as a bolometer for THz radiation detection [39,40]. When the resonator absorbs THz radiation, a temperature change causes the tuning of the resonance frequency. This frequency shift is proportional to the intensity of absorbed radiation. Due to the high-quality factor of MEMS resonators, very high sensitivity to small changes in absorbed radiation is provided. Moreover, since the thermal capacity of MEMS resonators is very small, high-speed THz detection is achievable. Depending on the geometrical shape and vibrational mode, they can be classified as bending mode, torsional mode, or membranous shape [41].

**Figure 16:**
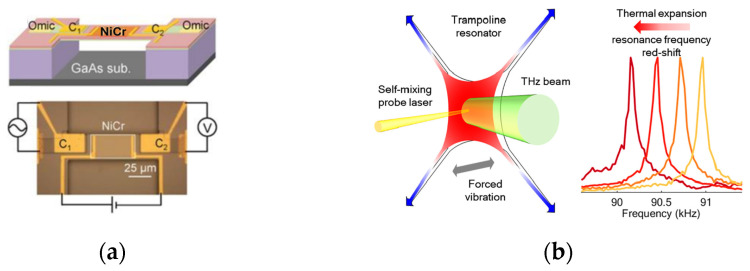
(**a**) A schematic view of a double-clamped MEMS beam resonator as a sensitive thermistor [39]; (**b**) an ultrasensitive micromechanical resonator bolometer using a Si_3_N_4_ trampoline [41].

Figure 16a shows a schematic view of a double-clamped MEMS beam resonator as a sensitive thermistor.

The support layer and the top gate electrodes on the two ends of the beam form two piezoelectric capacitors, *C*_1_ and *C*_2_. An AC voltage is applied to *C*_1_ to drive the beam, and *C*_2_ detects an induced oscillation signal. The upper and lower parts of the Figure 16a are two projections of one structure. The noise equivalent temperature difference is about 1 μK/√Hz), and *NEP* is ~90 pW/√Hz [39]. The bolometer is very sensitive and more than 100 times faster than other uncooled THz detectors, and its operation bandwidth is several kHz.

Another bolometer construction with an ultrasensitive MEMS resonator using a Si_3_N_4_ trampoline with a Cr–Au coating was presented in Figure 16b [41]. The applied small tether width minimizes heat losses. The trampoline’s vibration is read out using the self-mixing interferometric technique in which a 945 nm laser works as a probe [42]. In this setup, the light reflected by a vibrating trampoline is directed into the laser cavity. As a result, amplitude and frequency modulation of the optical beam is registered by a photodiode. In this way, low *NEP* values of 100 pW Hz^−1/2^ are provided.

The paper by D. Seliuta et al. describes another broadband thermal THz detector that utilizes a silicon lens-coupled asymmetric bow-tie diode based on an In_0.54_ Ga_0.46_ As layer [43]. Its spectral response ranged from 10 GHz to 2.52 THz at room temperature, and the voltage responsivity was 5 V/W below 1 THz. Therefore, it is useful for various applications, such as security imaging, medical diagnosis, and environmental monitoring.

**Figure 17:**
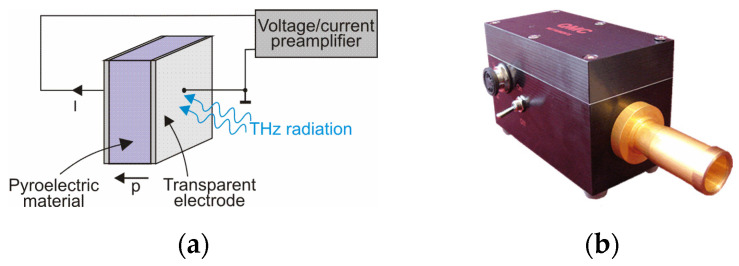
(**a**) Scheme of a pyroelectric detector; (**b**) QMC Instruments Ltd. (Cardiff, UK) room-temperature pyroelectric THz detector [44].

The pyroelectric detector belongs to the thermal detectors group and can work at room temperature. Its broad detection bandwidth, wide dynamic range, and high sensitivity levels make it one of the most commonly used THz detectors. It is fabricated in a capacitor-like structure consisting of a pyroelectric material sandwiched between two metal electrodes (Figure 17).

The materials used as pyroelectric uncooled THz detectors are lithium tantalate oxide (LiTaO_3_), lithium niobate oxide (LiNbO_3_), deuterated L-alanine doped tri-glycene sulfate (DLARGS), triglycine sulfate (TGS), deuterated triglycine sulfate (DTGS), and polyvinylidene difluoride (PVDF) [45,46]. One of the metal electrodes is coated with a black film to absorb the incoming terahertz radiation. The consequent heating of the film raises the temperature of the pyroelectric layer so that its polarization changes and a current is generated. The current responsivity of the detector is given by

(3) $$R_{i}=\frac{p\left(T\right)\alpha }{\rho c_{p}d},$$

and depends on the pyroelectrical coefficient (*p*) of the material, coefficient of radiation absorption (α), density of the crystal (*ρ*), specific heat (*c*_p_), and crystal thickness (*d*).

Reducing the crystal thickness and increasing the coating absorption will increase the current output and therefore improve the current responsivity of the pyroelectric detector. Next, the detector output signal is fed to a preamplifier.

Pyroelectric detectors optimized for operation in the wavelength range of 2–20 μm are characterized by a responsivity of 10^5^ V/W and noise equivalent power of 1 × 10^−9^ W/Hz^1/2^. Their performances drop at far-infrared and terahertz frequencies. It is caused by a decrease in absorption efficiency with the increase in radiation wavelength. The pyroelectric detector is relatively slow with a response time in the milliseconds range. The detector’s standard configuration can be equipped with an optical filtering window and black absorber to enhance flat spectral response.

Le et al. also developed a LiTaO_3_ pyroelectric array with a responsivity above 7 kV/W and *NEP* of 1.5 × 10^−9^ W/ Hz^1/2^ [47]. It was also shown that using the LiTaO_3_ and LiNO_3_ structures with a thickness of 25 µm, it is possible to obtain the current responsivity above 4 μA/W with *NEP* below 1.0 × 10^−10^ W/Hz^1/2^ [48].

It was also shown that the operating range may be extended to higher wavelengths by adding additional absorbers (absorptive metallic, oxide, or carbon nanotubes) to conventional IR pyroelectric detectors [49]. The metasurface absorbers enable near-to-perfect absorptivity within a narrow frequency band under the condition of *t*/*λ* << 1 (where *t* is the thin absorber). Such absorbers enable an adjustment of the spectral characteristics of the detectors through a proper modification of the metamaterial structure.

**Figure 18:**
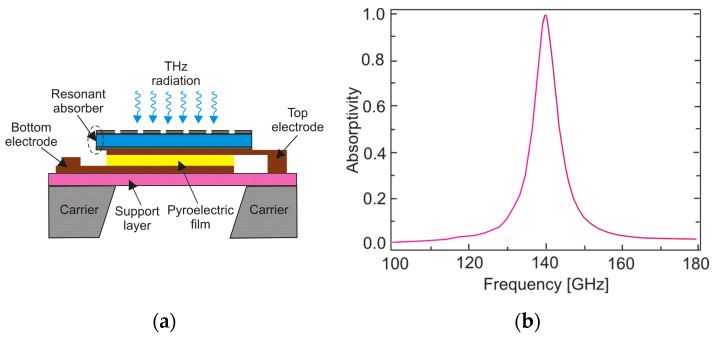
(**a**) Scheme of the pyroelectric detector with an integrated resonant absorber; (**b**) the absorptivity of the absorber [50].

Figure 18a shows a pyroelectric detector with an integrated resonant absorber. The absorber was combined with the commercial IR pyroelectric detector. This kind of detector exhibits voltage responsivity of 10^5^ V/W and *NEP* ~ 1.0 × 10^−9^ W/Hz^1/2^ [50]. Using the resonant metasurface absorber, the detection of longer wavelengths within a narrow spectral band centered at 140 GHz (*λ* = 2.14 mm) was ensured (Figure 18b). As a result, at the fundamental absorption frequency of 140 GHz, the voltage responsivity of the detector was 0.56 × 10^5^ V/W, while its *NEP* was estimated as 2.0 × 10^−9^ W/Hz^1/2^.

The above analysis showed that the parameters of thermal detectors are defined both by the selection of radiation-absorbing material, as well as the design of the temperature transducer itself and the methods of analyzing its output signal. As a summary, examples of the most interesting detectors’ parameters, described in this chapter, are listed in Table 2.

### 3.2. Application’s Study of THz Thermal Detectors

The development of thermal detectors made it possible to design devices that were applied in many THz photonics technologies used in, among others, medical imaging, security screening, non-destructive testing (NDT), communications, spectroscopy, chemical sensing, industrial process control, and biomedical and scientific research. These devices are mainly used to determine the level of radiation power or its spatial distribution.

As previously mentioned, both single detectors and matrices are used. In the first type of application, a single detector is mounted in a commercially available head that is very often equipped with electronics to read out and process the output signal from the detector. A single detector can also be used in an imaging device together with additional units enabling space scanning, e.g., a set of masks. Less complicated imagers use detector arrays integrated with an ROIC element. Comparing these imaging constructions, the scanning operation to obtain an image takes a long time. For example, the space corresponding to a 40 × 40-pixel frame will be scanned in more than 100 s. For matrix detectors, in principle, the frequency reaches several dozen Hz with a pixel size of 160 × 160. However, for very low levels of THz radiation, it is better to use scanning imagers that are characterized by lower noise levels and higher sensitivity.

**Figure 19:**
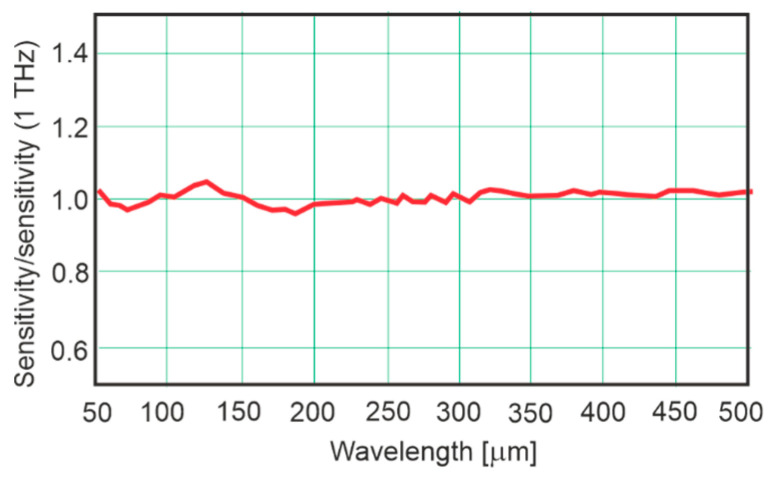
The sensitivity of the Lasertechnik pyroelectric detectors normalized to the sensitivity at 1 THz (adopted after [55]).

The performed literature analysis has shown that many articles and books thoroughly describe the working designs of single detectors or detector arrays in laboratories. However, there is no review of these technologies’ practical application, especially in the case of single detectors. Preliminary conclusions show that shelf-ready detectors are usually used in terahertz imaging and sensing systems. Currently, there are some THz detection heads on the market. In principle, their design does not differ from the heads used in the IR range, and the only difference is the type of absorber used to obtain not-so-low sensitivity in the THz range. For example, Figure 19 shows the sensitivity of the Lasertechnik company pyroelectric detectors normalized with the sensitivity at 1 THz. Between 200 and 500 µm the sensitivity changes within 2% [55].

**Figure 20:**
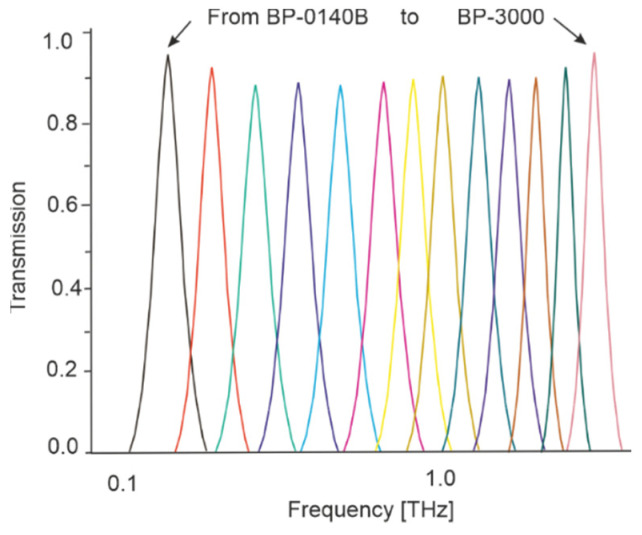
Examples of measured transmission spectra for some standard filters. BP means filter model, and the number means the central frequency (adopted after [55]).

Some detection heads can adjust their spectral sensitivity characteristics by using a set of dedicated spectral filters. For example, Figure 20 shows the transmission of some band-pass filters selecting specific frequencies in the terahertz waves.

Table 3 lists the parameters of some heads dedicated to the level-measuring of terahertz radiation.

Each commercial detector is meticulously prepared for strictly defined operating conditions. These conditions are crucial for the detector’s performance, and the properties of the detecting element and extra equipment and extraordinary designs determine them. Example operational conditions of the GC-1T detector are as follows: ambient operating pressure range of (760 ÷ 10^−3^) mm Hg, temperature range of (5 ÷ 40) °C, relative humidity of (0 ÷ 80)%, and the avoiding vibrations with a frequency of 1 ÷ 100 Hz [54]. Many methods are implemented into measuring devices with thermal detectors to reduce the impact of environmental factors. For stress or vibration compensation, micromechanical chip attachment or multilayer absorbers are used in a position that makes them mechanically similar so that the noise signal can be cancelled [56,57]. To overcome the impact of ambient temperature changes on these detectors, a special device construction with mechanical compensation, reference temperature detector (dummy detector), or active and passive thermal control can be applied. These methods ensure reliable and accurate detection, even in challenging environmental conditions [58,59,60,61].

**Figure 21:**
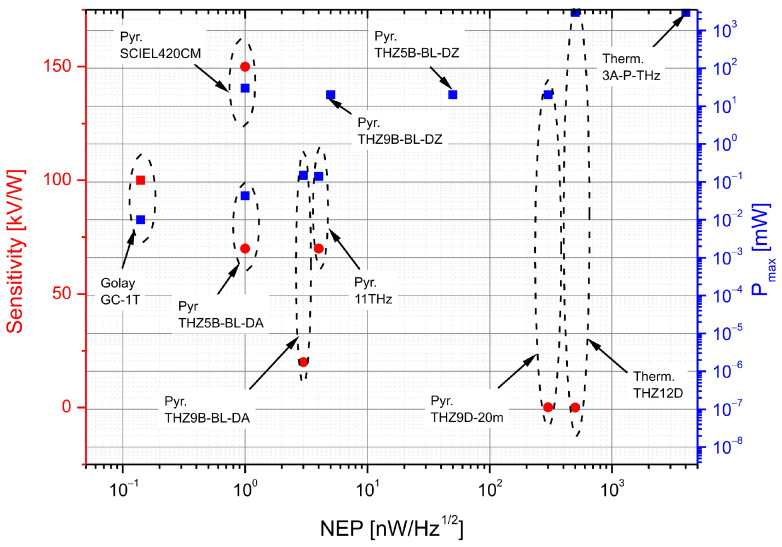
Parameters comparison of commercially available detection heads (red circles—sensitivity, blue squares—maximum power).

Figure 21 summarizes three parameters of detection heads: *NEP*, sensitivity, and maximum power (*P*_max_), which facilitates analysis and practical selection. It contains the parameters of the heads listed in Table 1, and others available on the market.

A similar parameters list was made for THz cameras (Table 4). In this type of device, as a thermal detector, microbolometers are the most frequently used.

The comparison also includes a modified pyroelectric camera that was previously dedicated to infrared research. For an imager with a single thermal detector, only one experimental design with the Golay cell has been reported.

Currently, THz detection systems can be used in most cases to monitor both cw and pulsed radiation sources. The exception is the pyroelectric detectors, which register only the radiation changes resulting from their principle of operation. Therefore, additional modulators should be used for cw radiation. These modulators could be external devices (e.g., for pyroelectric power meters) and integrated (for pyroelectric cameras). Electric motors or piezoelectric actuators often control mechanical modulators (choppers) in the low-frequency range. Technologies are also being developed that enable modulation rates above several hundred kHz, up to GHz [63]. For example, they use tunable metasurfaces combined with semiconductors, graphene superconductors, and 2D materials. In practice, it should be remembered that modulation of cw-detected radiation can significantly reduce the impact of background radiation and self-noise (minimization of 1/f-type noise and noise bandwidth). Therefore, modulation is also used for other thermal detectors to measure low-power radiation sources or minor changes in radiation levels (phase-sensitive detection) [7].

## 4. Conclusions

State-of-the-art THz thermal detectors show that they are an important tool used in many technologies related to scientific and industrial research, and increasingly in everyday life. Identification of these areas was carried out, during which the main criterion was not the type of detector and its construction, but the practical application in a specific THz system (Table 5). An important conclusion from this analysis is the fact that most of the thermal detectors used in these systems are already commercially available products. In most cases, these devices have only been modified for a given area of the entire system application.

**Figure 22:**
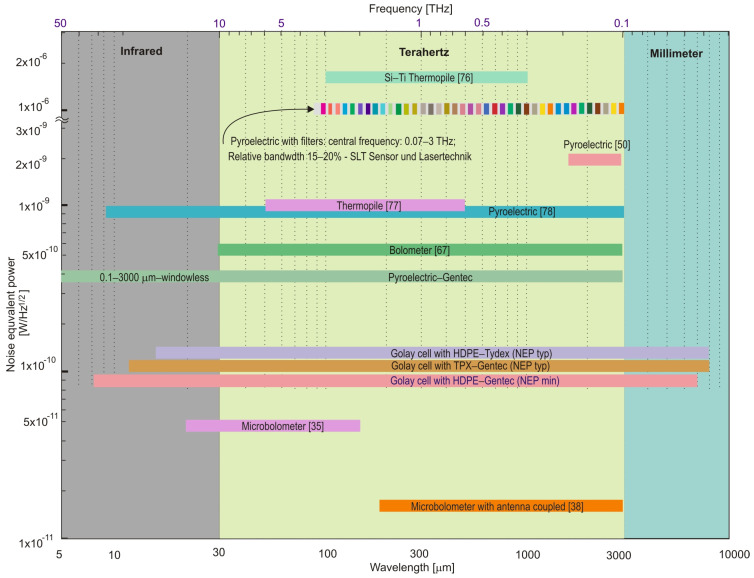
Spectral operation range of some THz thermal detectors. Refs. [35,38,50,67,76,77,78] are cited in the figure.

The main factor, influencing the interest in THz thermal detectors, is their wide characteristics of spectral sensitivity. A brief analysis of scientific works described in the literature was carried out and supplemented with data on commercially available detection heads. Figure 22 presents a unique summary of THz thermal detectors’ spectral operational ranges and NEP levels. Additionally, the transmission spectral ranges of the THz filters that can be used to match the detectors’ sensitivity spectrally are placed on the characteristics.

A review of previous research results has shown therefore that there is an ongoing effort to simultaneously meet the application requirements of a high operating rate and detection of very low signals in the presence of noise. These are important properties whose significant limitation is the principle of thermal processes, which are slower than photon ones. The directions of thermal detectors’ development include material technologies related to high-performance THz radiation absorbers, the design of temperature transducers with appropriate thermal interfaces, signal processing with, e.g., nano- or microstructures, and advanced detection methods.

There is still observed progress in the evolution of thermal room-temperature THz detectors. Further technological advancements resulting from the rapid development of THz detector technologies in wider practical applications can be expected.

## Figures and Tables

**Table 1:** Examples of the most-used absorber materials for terahertz applications.

Materials	Advantages	Drawbacks
Metals (Au, Ag, Cr, alloys)	Nanometric coatings Porous metal coatings (suitable for THz but not in IR)	Corrosive, poor wettability
Semiconductors (Si, Ge, GaAs)	Dopant/defect engineering Good thermal conductivity ^2^	Parasitic reflection ^1^ Difficult to machine in mm scale
Carbon (graphite, CNTs, graphene)	The variety of allotropic forms Good thermal conductivity ^2^	Complex synthesis ^2^, reproducibility/reliability, fragile
Dielectric (polymers)	Low cost, easy to shape	Thick (>4 mm at 0.1 THz) ^2,3^ Molecular vibrational absorption ^1^
Composite (polymer matrix)	Magnetic particles or any other fillers can be incorporated as a second phase	Inhomogeneity, reproducibility, low thermos-oxidative stability ^3^
Polymer-derived materials	Near net-shaped structures, High thermos-oxidative stability	High thermal mass (heat capacity) ^2^

^1^ Attenuator. ^2^ Thermal transducers. ^3^ Radar-absorbing materials.

**Table 2:** Examples of parameters of THz uncooled thermal detectors that are of greatest interest.

Detector Type	NEP [W/Hz^1/2^]	Responsivity [V/W]	Response Time [s]	Dynamic Range	Ref.
Pyroelectric	1 × 10^−9^	(0.05–3) × 10^5^	(10–50) × 10^−3^	-	[9]
	2 × 10^−9^	0.56 × 10^5^	2.3 × 10^−3^	-	[50]
	300 × 10^−9^ W ^(1)^	120	<0.2	P_max_ = 25 µW	[51]
Thermopile	8 × 10^−5^	35 nV/(W/m^2^)	200 × 10^−3^	-	[25]
	1.3 × 10^−11^	1.93 × 10^3^	2.5 × 10^−3^	~40 dB	[28] ^(6)^
	0.5 × 10^−6 (2)^	0.2	3	3 W ^(3)^	[51]
Bolometer	10^−10^	10^5^–10^6^	10^−3^	-	[52]
	4.57 × 10^−13^	2.18 × 10^3^	-	0.4–3.6 V ^(4)^	[35]
	1.4 × 10^−13^	300	10^−6^	10 µW–0.8 mW ^(5)^	[38]
Golay cell	1 × 10^−10^	1.8 × 10^3^ @3 THz	25 × 10^−3^	(16–600) µW	[53]
	0.8 × 10^−10^	10^5^	25 × 10^−3^	up to 100 µW	[54]

^(1)^ Power noise level. ^(2)^ Power noise level. ^(3)^ Maximum power level. ^(4)^ Output dynamic range of the readout integrated circuit. ^(5)^ Test conditions. ^(6)^ Thermocouple.

**Table 3:** Examples of parameters of some THz thermal detection heads for energy and power meters.

Model Company	11THZ Standa (Lithuania)	RM9-THz Ophir (USA)	THZ 9D Gentec (Canada)	GC-1T Tydex (Russia)	THZ 12D Gentec (Canada)
Rise time	0.2 s	3.5 s	<0.2 s	30 ms	3 s
Detector	Pyroelectric	Pyroelectric	Pyroelectric	Golay	Thermopile
Typical sensitivity	70 kV/W	x	120 V/W	1 × 10^5^ V/W	200 mV/W
Spectral range [THz]	0.1–30	0.1–30	0.1–30	0.04–23	0.1–30
Noise/NEP W/Hz^1/2^	4 × 10^−9^	20 × 10^−9^	300 × 10^−9^	1.4 × 10^−10^	0.5 × 10^−6^
Aperture	Dia. 5 mm	Dia. 8 mm	Dia. 9 mm	Dia. 8 mm	Dia. 12 mm
Min. power Max. power	x 140 µW	100 nW 100 mW	3 µW 20 mW	x 10 µW	50 µW 3 W
Max. power density	50 mW/cm^2^	5 W/cm^2^	3 W/cm^2^	x	30 W/cm^2^
Weight [kg]	0.5	0.2	0.091	0.8	0.316

(x—means no data available).

**Table 4:** Examples of parameters of some THz imaging devices.

Camera	i2S Vision CEA-Let	MICROXCAM-384i-THz ino.ca	Rigi Camera Rigi Swiss Terahertz	Pyrocam IV Ophir	Lab Setup [62]
View	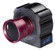	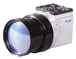	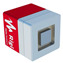	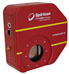	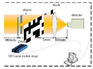
Spectral range	0.4–3 THz	0.094–4.25 THz	x	x	1.4–2.0 THz
Array	320 × 240 FPA Bolometer	384 × 288 FPA Bolometer	920 × 1080 FPA Bolometer	920 × 1080 FPA Pyroelectric	1× Golay cell (GC-1P)
Pixel pitch	50 µm	35 µm	from 15 µm	from 15 µm	1 mm
NEP	$\text{<}30\;\mathrm{pW}/\sqrt{Hz}$	$0.18\;\mathrm{pW}/\sqrt{Hz}$	$1.5\;\mathrm{pW}/\sqrt{Hz}$	$1.5\;\mathrm{pW}/\sqrt{Hz}$	x
Frame-rate	25 Hz	50 Hz	60 Hz	60 Hz	0.125 Hz
Camera size [cm]	12.5 × 11.5 × 6.5	6.1 × 6.1 × 6.5	3 × 3 × 4	14.7 × 14.7 × 5.5	x
Camera weight [kg]	x	0.36	<0.2	1.2	x

(x) No data.

**Table 5:** Parameters of the identified thermal detectors implemented in some THz systems.

Detector	THz System	Parameter	Ref.
Thermopile Oriel 3A-P with a calibrated absorber	Radiometer	Δ*f* = (0.1–30) THz *Min. power* = 15 µW τ = 2.5 s	[64]
Gollay cell	Fourier transform spectrometer	x	[65]
Pyroelectric Perkinelmer LHI778 with an optical filter	THz imaging	*R*_V_ = 1200 V/W *f* = 1.89 THz	[66]
Bolometer	Power meter	Δ*f* = (0.1–10) THz *NEP* = 50 nW/√Hz	[67]
Golay cell Tydex GC-1P	Power meter Δ*f* = (0.90 − 3.05) THz	Δ*f* = (0.04–20) THz *NEP* = 1.4 × 10^−10^ W/√Hz *R*_V_ = 10^5^ V∕W	[68]
Pyroelectric Gentec SPH-62 THz		Δ*f* = (0.1–30) THz *NEP* = 10^−9^ W/√Hz *R*_V_ = 7 × 10^4^ V∕W	
Bolometer MEMS	THz imaging	*R*_f_ = 23 Hz/µW *NEP* = 7.4 nW/√Hz	[69]
Pyroelectric	THz-TDS spectrometer	Δ*f* = (0.1–5) THz *R*_V_ = 160 V∕W *Min. power* = 1 µW	[70]
Pyroelectric LiTaO_3_	THz absorption spectrometer	*f* = 4.75 THz *R*_V_ = 70 kV/W *NEP* = 25 µW/√Hz	[71]
Bolometer		*R* = 42 MΩ *NEP* = 2.3 × 10^−17^ W/√Hz	
Golay cell Tydex TC-1T	THz imaging	*f* = 118 GHz	[72]
microbolometer array THz camera IR/V-T0831C NEC Corporation	Single-shot THz image	Δ*f* = (1–7) THz Format 320 × 240, 235 µm *NEP* < 100 pW/√Hz Frame rate = 8.5 Hz	[73]
Golay cell	Reflectometry/spectroscopy	Δ*f* = (0.2–1) THz	[74]
Pyroelectric array PYROCAM III	Terahertz holography	Δf = (0.1–300) THz Sensitivity 1.5 mW/cm^2^ Format 160 × 160, 75 µm *NEP* = 13 nW/√Hz	[75]

(x—means no data available).

## Data Availability

Data are contained within the article.

## References

[1] Rogalski A., Sizov F. (2011). Terahertz detectors and focal plane arrays. Opto-Electron. Rev..

[2] Li J., Li J. (2020). Terahertz (THz) Generator and Detection. Electr. Sci. Eng..

[3] Dhillon S.S., Vitiello M.S., Linfield E.H., Davies A.G., Hoffmann M.C., Booske J., Paoloni C., Gensch M., Weightman P., Williams G.P. (2017). The 2017 terahertz science and technology roadmap. J. Phys. D Appl. Phys..

[4] Assefzadeh M.M., Jamali B., Gluszek A.K., Hudzikowski A.J., Wojtas J., Tittel F.K., Babakhani A. Terahertz trace gas spectroscopy based on a fully-electronic frequency-comb radiating array in silicon. Proceedings of the CLEO 2016.

[5] Aji A.P., Apriono C., Rahardjo E.T. (2023). Input Power and Effective Area in Terahertz Detector Measurement: A Review. IEEE Access.

[6] Rieke G.H. (2003). Detection of Light: From Ultraviolet to the Submillimeter.

[7] Rogalski A., Bielecki Z. (2022). Detection of Optical Signals.

[8] Sizov F., Rogalski A. (2010). THz detectors. Prog. Quantum Electron..

[9] Sizov F. (2018). Terahertz radiation detectors: The state-of-the-art. Semicond. Sci. Technol..

[10] Rogalski A. (2019). Infrared and Terahertz Detectors.

[11] Niu Y., Wang Y., Wu W., Wen J., Cheng Y., Chen M., Jiang S., Wu D., Zhao Z. (2020). Efficient room-temperature terahertz detection via bolometric and photothermoelectric effects in EuBiTe3 crystal. Opt. Mater. Express.

[12] Müller R., Bohmeyer W., Kehrt M., Lange K., Monte C., Steiger A. (2014). Novel detectors for traceable THz power measurements. J. Infrared Millim. Terahertz Waves.

[13] Yan D., Wang Y., Qiu Y., Feng Q., Li X., Li J., Qiu G., Li J. (2022). A Review: The Functional Materials-Assisted Terahertz Metamaterial Absorbers and Polarization Converters. Photonics.

[14] Huang L., Chen H.T. (2013). A Brief review on terahertz metamaterial perfect absorbers. Terahertz Sci. Technol..

[15] Venkatachalam S., Zeranska-Chudek K., Zdrojek M., Hourlier D. (2020). Carbon-based terahertz absorbers: Materials, applications, and perspectives. Nano Sel..

[16] Rogalski A., Bielecki Z., Mikolajczyk J., Dakin J.P., Brown R.G.W. (2018). Detection of optical radiation. Handbook of Optoelectronics.

[17] Grzyb J., Pfeiffer U. (2015). THz Direct Detector and Heterodyne Receiver Arrays in Silicon Nanoscale Technologies. J. Infrared Millim. Terahertz Waves.

[18] Hillger P., Grzyb J., Jain R., Pfeiffer U.R. (2019). Terahertz Imaging and Sensing Applications with Silicon-Based Technologies. IEEE Trans. Terahertz Sci. Technol..

[19] Klocke D., Schmitz A., Soltner H., Bousack H., Schmitz H. (2011). Infrared receptors in pyrophilous (“fire loving”) insects as a model for new un-cooled infrared sensors. Beilstein J. Nanotechnol..

[20] Measuring THz Radiation: Choose a Pyroelectric Detector or Golay Cell?. https://www.photonicsonline.com/doc/measuring-thz-radiation-choose-a-pyroelectric-detector-or-golay-cell-0001.

[21] Application Note Gentec-eo, 201924_2014_V1.0. www.gentec-eo.com/Content/downloads/application-note/AN_201924_THz_R1.pdf.

[22] Desmarisa V., Rashida H., Pavolotskya A., Belitsky V. (2009). Design, simulations and optimization of micromachined Golay-cell based THz sensors operating at room temperature. Procedia Chem..

[23] Thermal Detectors. Hamamatsu Catalogue. https://www.hamamatsu.com/content/dam/hamamatsu-photonics/sites/documents/99_SALES_LIBRARY/ssd/thermopile_kird9005e.pdf.

[24] Huhn A.K., Spickermann G., Ihring A., Schinkel U., Meyer H.G., Bolívar P.H. (2013). Uncooled antenna-coupled terahertz detectors with 22 µs response time based on BiSb/Sb thermocouples. Appl. Phys. Lett..

[25] Mbarek S.B., Euphrasiea S., Barona T., Thierya L., Vairac P., Cretina B., Guillet J.-P., Chusseau L. (2013). Room temperature thermopile THz sensor. Sens. Actuators A Phys..

[26] Kašalynas I., Adam A.J.L., Klaassena T.O., Hoveniera N.J., Pandraudc G., Iordanov V.P., Sarro P.M. (2007). Some properties of a room temperature THz detection array. Advanced Optical Materials, Technologies, and Devices.

[27] Chen S.-J., Chen B. (2020). Research on a CMOS-MEMS Infrared Sensor with Reduced Graphene Oxide. Sensors.

[28] Varpula A., Timofeev A.V., Shchepetov A., Grigoras K., Hassel J., Ahopelto J., Ylilammi M., Prunnila M. (2017). Thermoelectric thermal detectors based on ultra-thin heavily doped single-crystal silicon membranes. Appl. Phys. Lett..

[29] Araki T., Li K., Suzuki D., Abe T., Kawabata R., Uemura T., Izumi S., Tsuruta S., Terasaki N., Kawano Y. (2023). Broadband Photodetectors and Imagers in Stretchable Electronics Packaging. Adv. Mater..

[30] Rogalski A. (2012). History of infrared detectors. Opto-Electron. Rev..

[31] Otsuji T. (2015). Trends in the Research of Modern Terahertz Detectors: Plasmon Detectors. IEEE Trans. Terahertz Sci. Technol..

[32] Khanna V.K. (2021). Bolometers, Golay cells and pyroelectric detectors. Practical Terahertz Electronics: Devices and Applications.

[33] Dem’yanenko M.A., Marchishin I.V., Startsev V.V. (2019). Absorption of terahertz radiation by a thin metal absorber in conventional and inverted bolometers. OSA Contin..

[34] Dem’yanenko M.A. (2018). Efficient broadband terahertz radiation detectors based on bolometers with a thin metal absorber. Tech. Phys..

[35] Gou J., Wang J., Zheng X., Gu D., Yu H., Jiang Y. (2015). Detection of terahertz radiation from 2.52 THz CO_2_ laser using a 320 × 240 vanadium oxide microbolometer focal plane array. RSC Adv..

[36] Gou J., Niu Q., Liang K., Wang J., Jiang Y. (2017). Frequency Modulation and Absorption Improvement of THz Micro-bolometer with Micro-bridge Structure by Spiral-Type Antennas. Nanoscale Res. Lett..

[37] Esfandiyari M., Lalbakhsh A., Jarchi S., Ghaffari-Miab M., Mahtaj H.N.R., Simorangkir B.V.B. (2022). Tunable terahertz filter/antenna-sensor using graphene-based metamaterials. Mater. Des..

[38] Kašalynas I., Venckevicius R., Minkevicius L., Sešek A., Wahaia F., Tamošiunas V., Voisiat B., Seliuta D., Valušis G., Švigelj A. (2016). Spectroscopic Terahertz Imaging at Room Temperature Employing Microbolometer Terahertz Sensors and Its Application to the Study of Carcinoma Tissues. Sensors.

[39] Zhang Y., Hosono S., Nagai N., Song S.H., Hirakawa K. (2019). Fast and sensitive bolometric terahertz detection at room temperature through thermomechanical transduction. J. Appl. Phys..

[40] Li C., Zang Y., Hirakawa K. (2023). Terahertz Detectors Using Microelectromechanical System Resonators. Sensors.

[41] Vicarelli L., Tredicucci A., Pitanti A. (2022). Micromechanical Bolometers for Subterahertz Detection at Room Temperature. ACS Photonics.

[42] Taimre T., Nikolic M., Bertling K., Lim Y.L., Bosch T., Rakic A.D. (2015). Laser feedback interferometry: A tutorial on the self-mixing effect for coherent sensing. Adv. Opt. Photonics.

[43] Seliuta D., Kasalynas I., Tamosiunas V., Balakauskas S., Martunas Z., Asmontas G., Valusis S., Lisauskas A., Roskos H.G., Kohler K. (2006). Silicon lens-coupled bow-tie InGaAs-based broadband terahertz sensor operating at room temperature. Electron. Lett..

[44] Terahertz Pyroelectric Detectors. https://www.terahertz.co.uk/qmci/thz-detector-systems/pyroelectric-detectors.

[45] Melnikov A.R., Kalneus E.V., Getmanov Y.V., Shevchenko D.A., Gerasimov V.V., Anisimov O.A., Fedin M.V., Veber S.L. (2023). Comparative Study of Single Crystal and Polymeric Pyroelectric Detectors in the 0.9–2.0 THz Range Using Monochromatic Laser Radiation of the NovoFEL. Polymers.

[46] Ohtake H., Suzuki Y., Kozeki T., Sarukura N., Ono S., Tsukamoto T., Nakanishi A., Nishizawa S., Stock M., Yoshida M., Sawchuk A. (2001). THz-radiation emitter and receiver system based on a 2-T permanent magnet, a 1.04-µm compact fiber laser, and a pyroelectric thermal receiver. Ultrafast Electronics and Optoelectronics.

[47] Li W., Wang J., Gou J., Huang Z., Jiang Y. (2015). Fabrication and Characterization of Linear Terahertz Detector Arrays Based on Lithium Tantalate Crystal. J. Infrared Millim. Terahertz Waves.

[48] Dooley D. (2010). Sensitivity of broadband pyroelectric terahertz detectors continues to improve. Laser Focus Word.

[49] Das D., Bhattacharyya K., Baruah S. (2020). A review of Terahertz technology and Metamaterial based electromagnetic absorber at Terahertz band. ADBU-J. Eng. Technol..

[50] Kuznetsov S.A., Paulish A.G., Navarro-Cía M., Arzhannikov A.V. (2016). Selective pyroelectric detection of millimeter waves using ultra-thin metasurface absorbers. Sci. Rep..

[51] User Manual THZ-D Series. Gentec-EO Inc. https://downloads.gentec-eo.com/prod/d2ba7bc4/103809-Manual-THZ-Series-Rev-1.7.pdf.

[52] Rogalski A. (2022). Progress in performance development of room temperature direct terahertz detectors. J. Infrared Millim. Terahertz Waves.

[53] Golay Cell Manual. SN: 160735. Microtech Instruments Inc. https://www.bnl.gov/atf/docs/goleycell_manual.pdf.

[54] Golay Detectors. TYDEX. https://www.tydexoptics.com/products/thz_devices/golay_cell.

[55] SLT Sensor– und Lasertechnik GmbH. https://www.pyrosensor.de/slt_katalog-gesamt-pdf-920267.pdf.

[56] Wu Q.Q., Zhao Y., Li X.C. (2024). Pyroelectric infrared device with multilayer compensation structure. Sens. Actuators A Phys..

[57] Mbarek S.B., Alcheikh N., Younis M.I. (2022). Recent advances on MEMS based Infrared Thermopile detectors. Microsyst. Technol..

[58] Rieh J.S. (2020). Introduction to Terahertz Electronics.

[59] Sebastián E., Armiens C., Gómez-Elvira J. (2011). Infrared temperature measurement uncertainty for unchopped thermopile in presence of case thermal gradients. Infrared Phys. Technol..

[60] Vybornov P. (2019). Prospects of uncooled metal bolometers. IEEE Photonics Technol. Lett..

[61] Liess M., Charlebois A., Hausner M., Ernst H., Karagözoglu H., Schilz J. (2006). Stabilization of the output signal of thermopile sensors in the thermal environment of automotive applications. Photonic Applications for Aerospace, Transportation, and Harsh Environments.

[62] Duan P., Wang Y., Xu D., Yan C., Yang Z., Xu W., Yao J. (2016). Single-pixel imaging with tunable terahertz parametric oscillator. Appl. Opt..

[63] Wang L., Zhang Y., Guo X., Chen T., Liang H., Hao X., Yang Z. (2019). A review of THz modulators with dynamic tunable metasurfaces. Nanomaterials.

[64] Steiger A., Kehrt M., Monte C., Müller R. (2013). Traceable terahertz power measurement from 1 THz to 5 THz. Opt. Express.

[65] Jiang Y., Liang M., Jin B., Kang L., Xu W., Chen J., Wu P. (2012). A simple Fourier transform spectrometer for terahertz applications. Chin. Sci. Bull..

[66] Yang J., Gong X.J., Zhang Y.D. (2009). Research of an infrared pyroelectric sensor based THz detector and its application in CW THz imaging. International Symposium on Photoelectronic Detection and Imaging 2009: Terahertz and High Energy Radiation Detection Technologies and Applications.

[67] Ling C.C., Rebeiz G.M. (1991). A wide-band monolithic quasi-optical power meter for millimeter-and submillimeter-wave applications. IEEE transactions on microwave theory and techniques. IEEE Trans. Microw. Theory Tech..

[68] Perenzoni M., Paul D.J. (2014). Physics and Applications of Terahertz Radiation.

[69] Morohashi I., Zhang Y., Qiu B., Irimajiri Y., Sekine N., Hirakawa K., Hosako I. (2020). Rapid Scan THz Imaging Using MEMS Bolometers. J. Infrared Millim. Terahertz Waves.

[70] Globisch B., Dietz R.J., Göbel T., Schell M., Bohmeyer W., Müller R., Steiger A. (2015). Absolute terahertz power measurement of a time-domain spectroscopy system. Opt. Lett..

[71] Wubs J.R., Macherius U., Lü X., Schrottke L., Budden M., Kunsch J., Weltmann K.-D., van Helden J.-P.H. (2024). Performance of a High-Speed Pyroelectric Receiver as Cryogen-Free Detector for Terahertz Absorption Spectroscopy Measurements. Appl. Sci..

[72] Takan T., Özkan V.A., İdikut F., Yildirim I.O., Şahin A.B., Altan H. (2014). Compressive sensing imaging through a drywall barrier at sub-THz and THz frequencies in transmission and reflection modes. Image and Signal Processing for Remote Sensing XX.

[73] Yue Z., Peng X., Li G., Zhou Y., Pu Y., Zhang Y. (2024). Single-Shot Direct Transmission Terahertz Imaging Based on Intense Broadband Terahertz Radiation. Sensors.

[74] Bennett D.B., Li W., Taylor Z.D., Grundfest W.S., Brown E.R. (2010). Stratified media model for terahertz reflectometry of the skin. IEEE Sens. J..

[75] Rong L., Latychevskaia T., Chen C., Wang D., Yu Z., Zhou X., Li Z., Huang H., Wang Y., Zhou Z. (2015). Terahertz in-line digital holography of human hepatocellular carcinoma tissue. Sci. Rep..

[76] Mbarek S.B., Euphrasie S., Baron T., Thiery L., Vairac P., Briand D., Guillet J.P., Chusseau L. (2015). Room temperature Si–Ti thermopile THz sensor. Microsyst. Technol..

[77] Kasalynas I., Adam A.J.L., Klaassen T.O., Hovenier J.N., Pandraud G., Iordanov V.P., Sarro P.M. (2008). Design and Performance of a Room-Temperature Terahertz Detection Array for Real-Time Imaging. IEEE J. Sel. Top. Quantum Electron..

[78] Berry C.W., Yardimci N.T., Jarrahi M. (2014). Responsivity Calibration of Pyroelectric Terahertz Detectors. arXiv.

